# In and out of the minor groove: interaction of an AT-rich DNA with the drug CD27

**DOI:** 10.1107/S139900471400697X

**Published:** 2014-05-24

**Authors:** Francisco J. Acosta-Reyes, Christophe Dardonville, Harry P. de Koning, Manal Natto, Juan A. Subirana, J. Lourdes Campos

**Affiliations:** a Departament d’Enginyeria Química, Universitat Politécnica de Catalunya, Diagonal 647, 08028 Barcelona, Spain; b Instituto de Química Médica, IQM–CSIC, Juan de la Cierva 3, 28006 Madrid, Spain; c Institute of Infection, Immunity and Inflammation, College of Medical, Veterinary and Life Sciences, University of Glasgow, 120 University Place, Glasgow G12 8TA, Scotland

**Keywords:** CD27, minor-groove binding drug, AT-rich DNA, d(AAAATTTT)_2_

## Abstract

The DNA of several pathogens is very rich in AT base pairs. Typical examples include the malaria parasite *Plasmodium falciparum* and the causative agents of trichomoniasis and trypanosomiases. This fact has prompted studies of drugs which interact with the minor groove of DNA, some of which are used in medical practice. Previous studies have been performed almost exclusively with the AATT sequence. New features should be uncovered through the study of different DNA sequences. In this paper, the crystal structure of the complex of the DNA duplex d(AAAATTTT)_2_ with the dicationic drug 4,4′-bis(imidazolinylamino)diphenylamine (CD27) is presented. The drug binds to the minor groove of DNA as expected, but it shows two new features that have not previously been described: (i) the drugs protrude from the DNA and interact with neighbouring molecules, so that they may act as cross-linking agents, and (ii) the drugs completely cover the whole minor groove of DNA and displace bound water. Thus, they may prevent the access to DNA of proteins such as AT-hook proteins. These features are also expected for other minor-groove binding drugs when associated with all-AT DNA. These findings allow a better understanding of this family of compounds and will help in the development of new, more effective drugs. New data on the biological interaction of CD27 with the causative agent of trichomoniasis, *Trichomonas vaginalis*, are also reported.

## Abstract 2

New features of an antiprotozoal DNA minor-groove binding drug, which acts as a cross-linking agent, are presented. It also fills the minor groove of DNA completely and prevents the access of proteins. These features are also expected for other minor-groove binding drugs when associated with suitable DNA targets.

## 1. Introduction   

**Figure 1:**
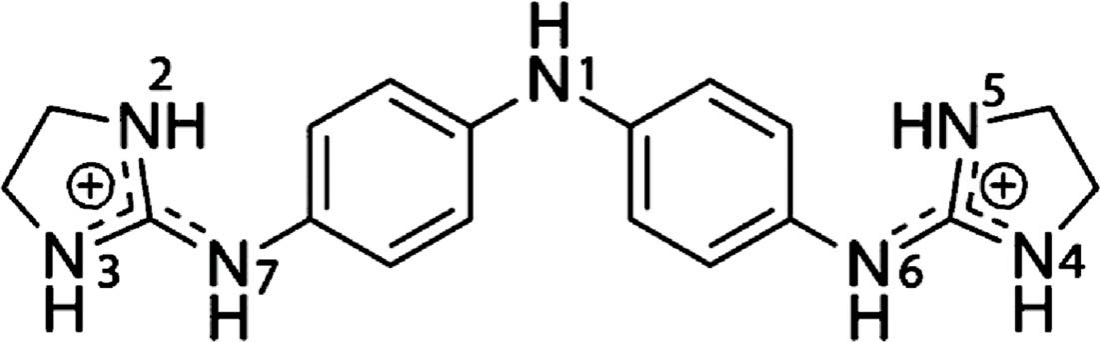
Chemical structure of CD27.

Enormous progress has been achieved in the past in the study of small-molecule ligands that have affinity for the DNA minor groove, as recently reviewed by Sheng *et al.* (2013 ▶). More complex types of drug binding to DNA have also been reviewed by Boer *et al.* (2009 ▶). Dervan and coworkers have carried out an extensive series of studies (Sheng *et al.*, 2013 ▶; Chenoweth & Dervan, 2009 ▶) aimed at developing ligands that recognize specific DNA sequences. Some intercalating drugs also favour binding through the minor groove (Niyazi *et al.*, 2012 ▶). The main group of studies has concentrated on the interaction of different drugs with AT-rich DNA regions, mainly with the Dickerson–Drew dodecamer d(CGCGAA­TTCGCG), which easily provides crystals with high resolution suitable for X-ray analysis. However, there is no evidence that AATT is the preferred sequence of interaction *in vivo*. In fact, little is known of the eventual DNA-sequence selectivity. We therefore decided to study the interaction of the all-AT DNA sequence d(AAAATTTT) with the dication 4,4′-bis(imidazolinylamino)diphenylamine (CD27; shown in Fig. 1 ▶).

CD27 is chemically related to diamidines, a class of dicationic DNA minor-groove binders with a long history of clinical success as antiprotozoal agents (Paine *et al.*, 2010 ▶; Soeiro *et al.*, 2005 ▶). In the last few years, our group has discovered a set of similar related compounds (*i.e.* bisimidazolinium diphenyl compounds) that kill African trypanosomes, the aetiological agent of sleeping sickness, very efficiently *in vitro* (Dardonville & Brun, 2004 ▶). In addition, some of these compounds were also very active *in vitro* against the malaria parasite *Plasmodium falciparum* (Rodríguez *et al.*, 2008 ▶). CD27, in particular, proved to be a potent inhibitor of *Trypanosoma brucei* growth *in vitro* (Dardonville & Brun, 2004 ▶) and *in vivo*. This compound was able to cure 100% of mice in the STIB900 murine model of stage 1 sleeping sickness (Dardonville *et al.*, 2006 ▶), but was not effective in the late (CNS) stage of the illness owing to poor blood–brain barrier permeability (Nieto *et al.*, 2011 ▶). Here, we provide additional evidence of its antiprotozoal activity by demonstrating a growth-inhibiting effect on *Trichomonas vaginalis* parasites, the pathogens responsible for the most common sexually transmitted infection in the world (Johnston & Mabey, 2008 ▶). *T. vaginalis* is a monogenetic, anaerobic, amitochondrial parasite, and as such is very different from kinetoplastid parasites such as *Trypanosoma* species, which are digenetic, aerobic and have functional mitochondria that perform essential functions. Despite these differences, the genomes of these parasites have in common a high content of AT base pairs. Thus, it was of interest to assess the effects of bis­imidazolines on *T. vaginalis* and compare this with the effects on trypanosomes, apart from the inherent interest in new drug leads against this major human pathogen (Johnston & Mabey, 2008 ▶).

The DNA-binding properties of CD27 have previously been studied using different techniques such as thermal melting curves [*T*_m_ = 38.5° for poly(dAdT)_2_; Dardonville *et al.*, 2006 ▶)], fluorescence intercalator displacement (FID) and biosensor surface plasmon resonance (SPR; Glass *et al.*, 2009 ▶). The crystal structure of CD27 bound to the self-complementary nucleotide d(CTTAATTCGAATTAAG)_2_ has previously been determined using a host–guest approach (Glass *et al.*, 2009 ▶). The compound was found to interact with the two central AATT sequences in a similar way to that found in other drugs which interact with the Dickerson–Drew dodecamer.

In the current paper, we describe a completely different DNA interaction behaviour of CD27: the compound completely covers the minor groove of the two A-tracts of the oligonucleotide d(AAAATTTT)_2_. Moreover, we found that the drug may interact with a neighbouring DNA molecule. These results show the need to study the interaction of drugs with the minor groove of different AT-rich sequences. In this sense, we have recently reported striking results for the interaction of pentamidine with an alternating AT oligo­nucleotide (Moreno *et al.*, 2010 ▶).

## 2. Materials and methods   

### 2.1. Synthesis   

The deoxyoligonucleotide d(AAAATTTT) was synthesized at the Pasteur Institute as the ammonium salt on an automatic synthesizer by the phosphoramidite method. It was purified by gel filtration and reverse-phase HPLC.

The synthesis of *N* ^1^-(4,5-dihydro-1*H*-imidazol-2-yl)-*N* ^4^-{4-[(4,5-dihydro-1*H*-imidazol-2-yl)amino]phenyl}benzene-1,4-diamine (CD27) was performed following a procedure described previously (Dardonville *et al.*, 2000 ▶). Two equivalents of *N*,*N*′-di(*tert*-butoxycarbonyl)imidazolidine-2-thione were reacted with one equivalent of 4,4-diaminodiphenyl­amine in the presence of mercuric chloride and an excess of triethylamine in dimethylformamide to give, after purification by silica chromatography, the corresponding fully Boc-protected derivative of CD27: tetra-*tert*-butyl 2,2′-{[azanediylbis(4,1-phenylene)]bis(azanediyl)}bis(imidazolidine-1,3-dicarboxylate). Removal of the Boc protecting groups was carried out with an HCl_gas_-saturated solution of dioxane at room temperature overnight. Recrystallization from methanol/diethyl ether yielded pure CD27 as the dihydro­chloride salt.

### 2.2. Biological assays   

The assays used in order to determine the effect of CD27 and related drugs on *Trichomonas* species are given in the Supporting Information^1^. In brief, compound susceptibility was tested using either the fluorescent dye resorufin as an indicator of viability (only live cells metabolize it to the nonfluorescent dihydroresorufin) or the fluorophore propidium iodide (to measure cell numbers based on the binding of propidium iodide to their DNA and RNA) (Natto *et al.*, 2012 ▶).

### 2.3. Crystallization   

The crystals were grown by vapour diffusion at 15°C using the hanging-drop method. We explored various divalent cations for crystallization by DLS (dynamic light scattering) and found that Mn^2+^ was the most appropriate. A detailed report is given in the Supporting Information. Thus, we used the following conditions: a pre-incubated DNA–CD27 complex in sodium cacodylate buffer was added to a drop with final concentrations of 0.25 m*M* DNA duplex, 0.75 m*M* CD27, 40 m*M* sodium cacodylate buffer pH 6.5, 8 m*M* MnCl_2_, 0.5 m*M* spermine and 5% MPD and equilibrated against a 30% MPD reservoir. MPD acts both as a precipitant and a cryoprotectant. After two weeks, polyhedral crystals appeared (shown in the Supporting Information).

### 2.4. Data collection and structure determination   

The crystals were flash-cooled at −173°C. A PILATUS 6M detector on beamline BL13-XALOC at the ALBA synchrotron was used for data collection, at a wavelength of 0.979 Å, to a maximum resolution of 2 Å. A summary of crystal data and refinement statistics is given in Table 1 ▶. The data were integrated using *XDS* (Kabsch, 2010 ▶) and were scaled using *SCALA* (Evans, 2006 ▶). The space group turned out to be hexagonal, as confirmed using *POINTLESS* (Evans, 2006 ▶), which indicated *P*6_1_22 and *P*6_5_22 as possible space groups. A theoretical B-DNA model was constructed using *TURBO-FRODO* (http://www.afmb.univ-mrs.fr/-TURBO-), with a base-pair stacking of 3.25 Å, a uniform base-pair twist of 36° and Watson–Crick base-pair bonding. It was used as a starting search model for molecular replacement, but without success even after an exhaustive search in different hexagonal and monoclinic space groups. Since the diffraction pattern showed three orientations of stacked oligonucleotides crossed by about 60° to their neighbours, we generated a possible arrangement of the oligonucleotides in the crystal with these requirements: we built another theoretical search model formed by a column of three B-DNA duplexes which had a −26° virtual twist between terminal base pairs (Campos *et al.*, 2006 ▶). A final solution was only obtained after using the column of three duplexes as a search model in the monoclinic space group *C*121 (*a* = 135.29, *b* = 78.10, *c* = 90.54 Å, β = 90.02°), where the asymmetric unit is formed by three columns of duplexes. In the first round of molecular replacement we could place one column using *Phaser* (McCoy *et al.*, 2007 ▶). This replacement was refined with *REFMAC*5 (Murshudov *et al.*, 2011 ▶). Firstly, a rigid-body refinement was performed up to 3.4 Å resolution with duplexes defined as groups. After a few cycles of maximum-likelihood isotropic restrained refinement with Watson–Crick hydrogen-bond distances restrained, the obtained model was used as a search model for molecular replacement with *Phaser*. Thus, it was possible to place the three columns (nine duplexes) of the full asymmetric unit in the correct position. In this model the hexagonal screw axis symmetry and its direction were clear. Thus, the correct space group is *P*6_1_22 (*a* = *b* = 78.05, *c* = 91.66 Å). Before translating the solution to the hexagonal space group the drug was placed in the minor groove with two drugs per duplex using *Coot* (Emsley *et al.*, 2010 ▶). The drug coordinates from a previous structure (PDB entry 3fsi; Glass *et al.*, 2009 ▶) were not suitable since they contained several anomalous bonds and angles. Therefore, a stereochemical restraint dictionary was generated for CD27 with the help of the *Grade* web server (http://grade.globalphasing.org). The values obtained were confirmed by an *ab initio* calculation (at B3LYP/6-311++G*) and by comparison with related compounds in the Cambridge Crystallographic Database (http://www.ccdc.cam.ac.uk). The final model was formed by one and a half DNA duplexes and three CD27 molecules. Using this model, molecular replacement with *Phaser* led us to the correct placement of the final model. A stereochemical restraint dictionary was generated for CD27 with the help of the *Grade* web server. Several cycles of maximum-likelihood isotropic restrained refinement were performed using *REFMAC*5 to 2.1 Å resolution, with Watson–Crick hydrogen-bond distances restrained. Noncrystallographic symmetry (NCS) was defined between single strands of DNA, jelly body set to 0.01. The external *Grade* CIF dictionary was used. For the last round of refinement the NCS was turned off and the correlation factors decreased to final values of *R*_work_ = 0.236 and *R*_free_ = 0.251 in the resolution range 20–2.1 Å, with a completeness of 97% (a 5% set of free reflections was used as an independent cross-validation indicator of the progress of refinement). No divalent ions were detected. Solution coordinates have been deposited in the Protein Data Bank as PDB entry 4ocd. The DNA structural parameters were analyzed with the help of the 3*DNA* software (http://x3dna.org/). Drawings were prepared with *PyMOL* (http://www.pymol.org).

## 3. Results   

### 3.1. Effects of bisimidazolines on *T. vaginalis*   

Compound CD27 and two closely related analogues were tested *in vitro* against the human pathogen *T. vaginalis* using two different protocols: the resorufin and the propidium iodide (PI) assays, respectively (Natto *et al.*, 2012 ▶). In general, these compounds showed weak or no activity against *T. vaginalis*. However, CD27 displayed the lowest EC_50_ of the series, whereas its guanidine analogue CD25 was approximately twofold less active against this pathogen. The opposite results were obtained against *Trypanosoma brucei rhodesiense*, as shown in Table 2 ▶. Taken together, the high anti-*T. brucei* activity and the weak anti-trichomonal activity of these compounds are completely consistent with kinetoplastid DNA targeting (or at least a mitochondrial target) being more important than nuclear DNA. The AT-rich minicircles in kinetoplasts (Jensen & Englund, 2012 ▶) appear to be the target for drug interaction, given their unique structural features. This is in part driven by the strong accumulation of cations in the mitochondria of trypanosomes because of the mitochondrial membrane potential (Ibrahim *et al.*, 2011 ▶).

### 3.2. Structure of the complex   

**Figure 2:**
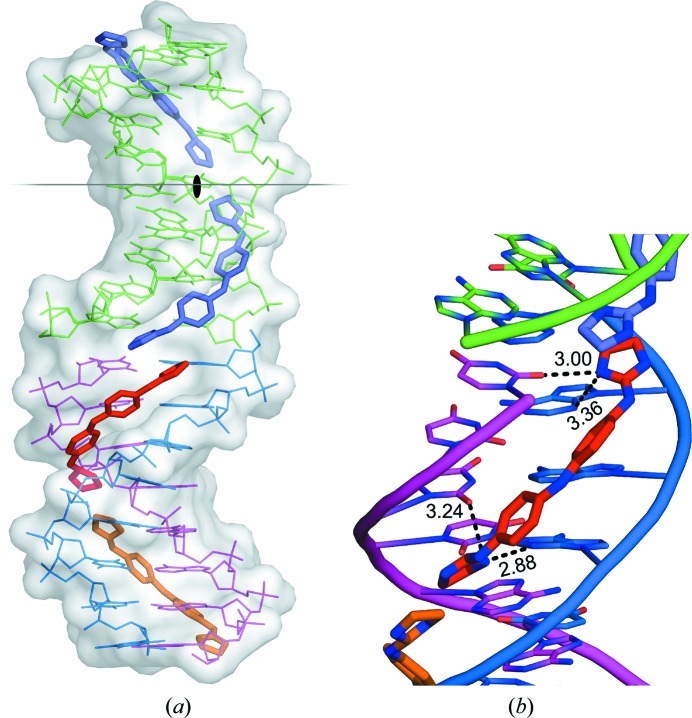
(*a*) View of the different crystallographic units of the complex. The black lozenge indicates one of the dyad axes. There are three independent oligonucleotide chains. One of them (green) forms a duplex with an identical symmetric chain. The other two (blue and magenta) form another DNA duplex. Three crystallographically independent drug molecules are indicated in different colours. (*b*) Hydrogen bonds formed by one CD27 drug with minor-groove atoms of the DNA duplex. N atoms of the drug are shown in dark blue. All three drugs show similar interactions.

The drug–DNA complex crystallized in a *P*6_1_22 unit cell with an asymmetric unit which contained three drugs plus one and a half duplexes, as shown in Fig. 2 ▶(*a*). Views of the unit cell are given in the Supporting Information.

The duplexes are stacked and organized as infinite continuous columns which cross in space at 60°. The drug molecules completely fill the minor groove of the DNA duplexes. No water molecules remain in the minor groove. Thus, the complex appears as a pseudo-continuous triple helix with one drug and two phosphodiester strands.

**Figure 3:**
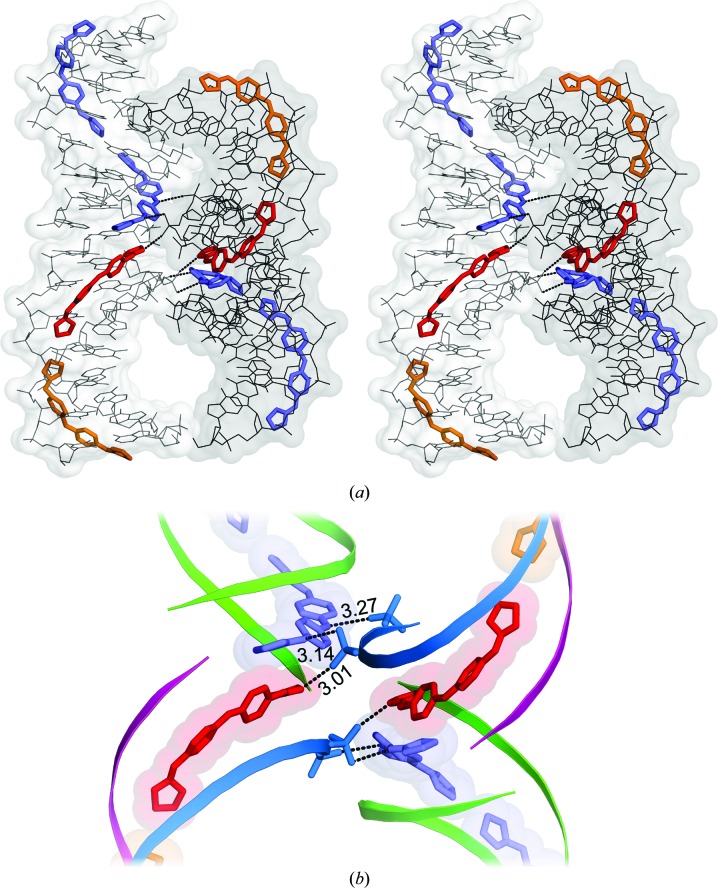
(*a*) Stereoview of crossed oligonucleotide duplexes, showing the interaction of two drugs with neighbouring phosphates of a symmetrical DNA chain. (*b*) Enlarged view of the interaction of two drugs with the neighbouring phosphates of a symmetrical DNA chain. A dyad axis runs through the centre of the figure.

As shown in the Supporting Information, the DNA–drug columns cross in space and are surrounded by large solvent channels. Crossings are stabilized in part by the interaction of the drug molecules with neighbouring DNA phosphates. Such interactions are shown in Fig. 3 ▶. A network of associated water molecules is also present in this region (not shown) and contributes to the stability of the crossings of DNA duplexes through phosphate–water–phosphate bridges. Drug *F* (orange) and DNA chain *C* (magenta) do not participate in the interactions: they face a solvent space in the crystal.

### 3.3. Drug conformation and interactions   

**Figure 4:**
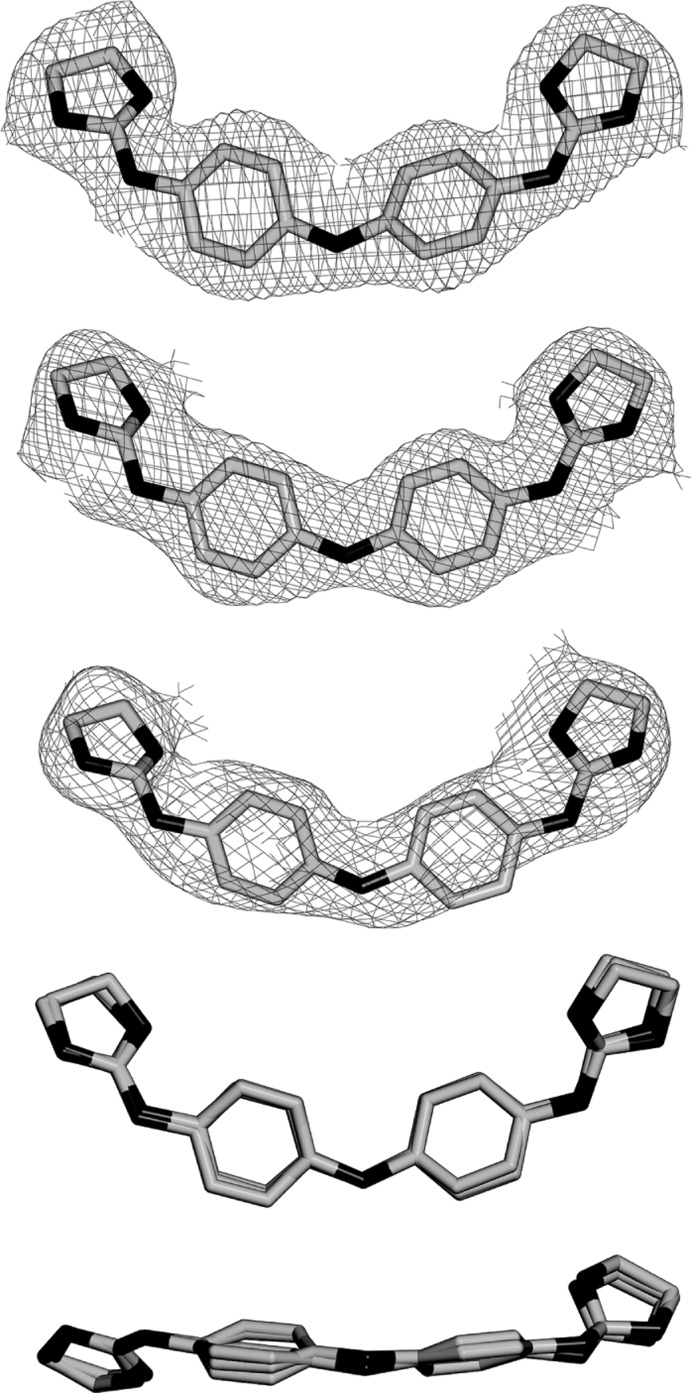
OMIT 2*F*_o_ − *F*_c_ electron-density map of the three drugs in the complex at the 1σ level. *D* is at the top, followed by *E* and *F* below. The bottom two frames show a superposition of the three drugs in two perpendicular views.

The three crystallographically independent CD27 molecules have very similar conformations, as shown in Fig. 4 ▶, with maximum r.m.s. differences of 0.17 Å. Interestingly, they interact with the A-tracts and not with the central AATT sequence. In most previous studies (Sheng *et al.*, 2013 ▶), minor-groove binding drugs were found in association with the GAATTC sequence. All three CD27 drugs form tight van der Waals interactions with the minor groove of DNA along the whole molecule. They have clearly different ends, in spite of the fact that CD27 has a symmetrical chemical structure (Fig. 1 ▶). On the one hand, the imidazoline rings placed in the centre of the duplexes show van der Waals interactions through their coplanar edges, with distances in the range 3.6–4.0 Å. On the other hand, the imidazoline rings at the other end of the molecule are placed in the terminal region of each DNA duplex: they are π-stacked with the terminal imidazoline of the CD27 neighbour. The charged terminal imidazoline groups also interact with different bases in the minor groove, as shown in Fig. 2 ▶(*b*) and Table 3 ▶. They form bifurcated hydrogen bonds with thymine and adenine atoms in opposite DNA strands.

The central amino group of the drug always faces away from the DNA. In molecule *D* it interacts with a phosphate from a neighbouring DNA molecule, as shown in Fig. 3 ▶. In the case of molecule *E*, it is associated with a water molecule in the solvent.

As we have just shown, the three independent CD27 molecules present very similar features. However their interactions with neighbouring phosphates are different. Drug *D* interacts with two phosphates, whereas drug *E* interacts with only one. Drug *F* has no external interactions. As a result the latter has higher *B* factors and is more disordered, as can be appreciated in Fig. 4 ▶. These differences are mainly owing to their different positions in the crystal. Molecule *F* faces a solvent region and no external interactions are possible.

### 3.4. DNA structure   

The two crystallographically independent duplexes in the structure are very similar, with an r.m.s. difference of 0.49 Å between the two duplexes (*A*–*A* and *B*–*C*). The duplexes show the standard features of A·T base pairs, with an average propeller twist of −13.6°. The duplexes are rather straight; the roll angles of individual steps have values of below 5°. This was confirmed using the *CURVES* program (Lavery *et al.*, 2009 ▶), which shows only a slight curvature in the case of the *B*–*C* duplex. The strong bending found in solution for this sequence (Stefl *et al.*, 2004 ▶) is absent in our crystal structure. The presence of the drug in the minor groove probably restricts the bending of the duplex.

An unexpected feature of the DNA structure is the high twist value in the AA/TT base steps, which have an average value of 38.5°. In previous structures an average of 35° was reported (Gorin *et al.*, 1995 ▶; Subirana & Faria, 1997 ▶). The difference is probably owing to the fact that the latter average included mainly AATT sequences, which have low twist values. High values are also observed in other structures that have AAA sequences (Edwards *et al.*, 1992 ▶; Valls *et al.*, 2005 ▶). Thus, we can conclude that long adenine stretches will have a high value of twist, which may explain some of the anomalous features observed in A tracts.

**Figure 5:**
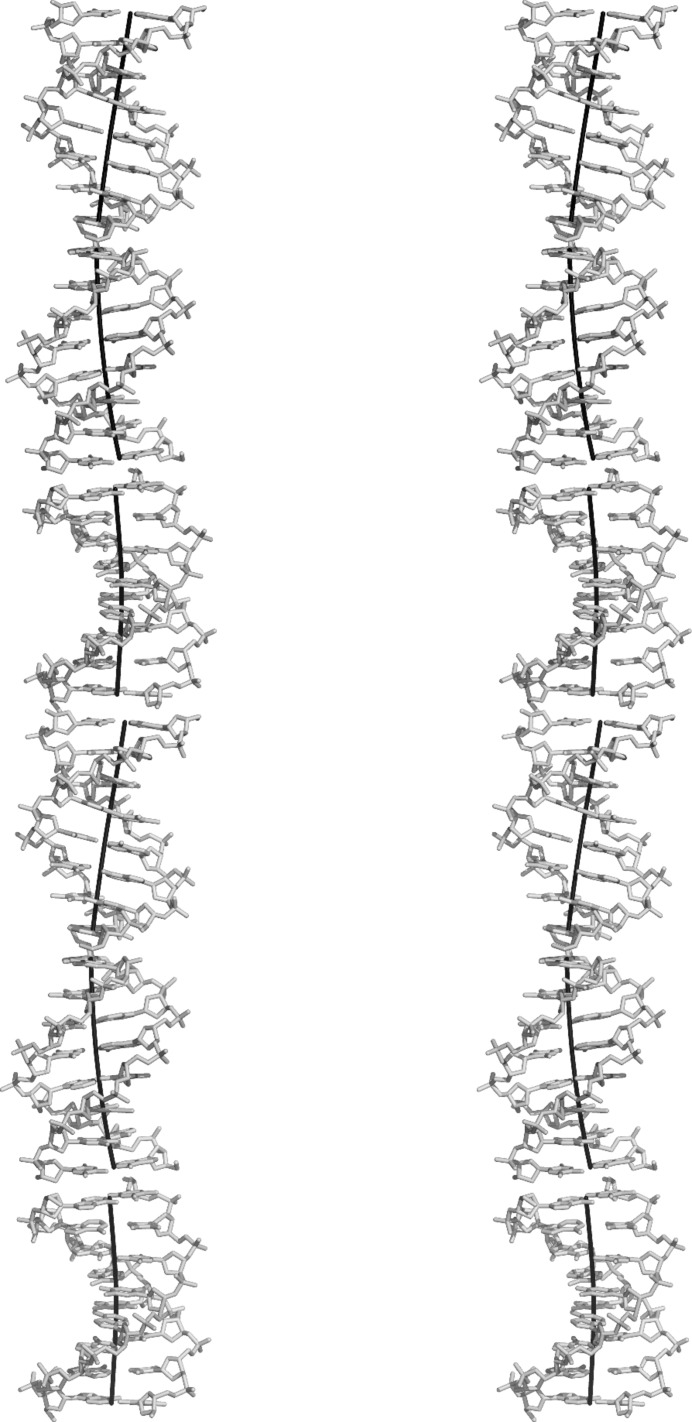
Stereoview of the helical organization of the duplex columns in the crystal. The axis of each individual duplex is also indicated (calculated with *CURVES*). The drug is not shown.

The duplexes in our structure are organized as infinite columns, as shown in Fig. 5 ▶. They are similar to those described for other all-AT octamer duplexes (Valls *et al.*, 2005 ▶; Campos *et al.*, 2006 ▶). Thus, the value of twist in the base step TA between terminal bases of neighbouring duplexes is negative (−26°). In our case there are three duplexes in the repeating unit, so that the average rotation angle Ω between neighbouring duplexes is 240°. In other octamers (Valls *et al.*, 2005 ▶; Campos *et al.*, 2006 ▶) it is smaller at close to 230°. Another feature of these columns is that the angle of the axis of each duplex with respect to the overall axis of the column is 9°. Thus, the duplexes are organized as a smooth coiled coil (De Luchi *et al.*, 2011 ▶), as shown in Fig. 5 ▶.

## 4. Discussion   

In the present study, we have found that the CD27 molecule completely covers the entire minor groove of the DNA duplexes. Since the duplexes are stacked in columns, the complex appears as a continuous triple helix formed by two single strands of DNA and one strand of CD27 molecules arranged end to end (Fig. 2 ▶ *a*). To our knowledge, complete coverage of the minor groove has not been described previously, with the exception of the complex of the unusual duplex d(CCCCCIIIII)_2_ with netropsin (Chen *et al.*, 1998 ▶).

**Figure 6:**
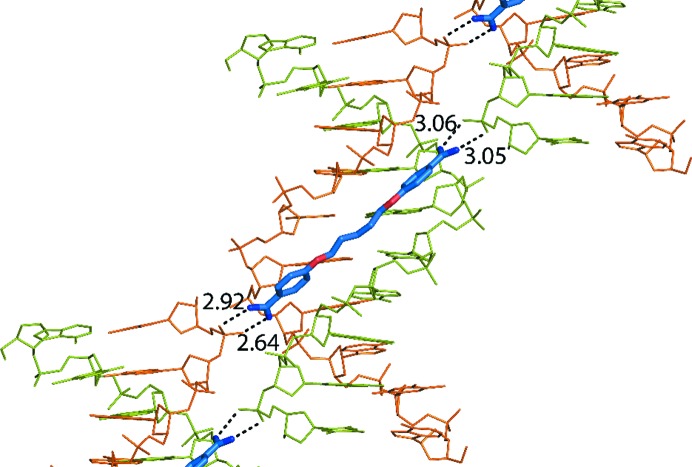
Interaction of pentamidine with neighbouring molecules in a complex with DNA (Moreno *et al.*, 2010 ▶). Hydrogen bonds between the terminal N atoms of pentamidine and phosphates are indicated.

Another unique feature of the complex is the interaction of CD27 with the phosphates of neighbouring molecules in the crystal, as shown in detail in Fig. 3 ▶. Interactions are found both in the terminal charged groups of CD27 and in its central N1 atom. Similar features have been observed (Moreno *et al.*, 2010 ▶) in the complex formed by pentamidine and the alternating duplex d(ATATATATAT)_2_. A scheme of the inter­actions is presented in Fig. 6 ▶. Such interactions are allowed by crystal packing. In contrast, in the studies performed with the conventional Dickerson dodecamer d(CGCGAATTCGCG)_2_ (Sheng *et al.*, 2013 ▶) no external interactions are found since the drug is always completely buried inside the minor groove. In this case, crystal packing also prevents interaction of the drugs with neighbouring molecules. The interactions with neighbouring phosphates that we have described are certainly a feature of this sequence, and demonstrate that minor-groove binding drugs may interact with neighbouring molecules, including other DNA duplexes. It is likely that other drugs might show similar interactions when bound to appropriate DNA sequences. The formation of cross-links may be a feature related to their biological action.

## 5. Conclusions   

Our studies show two new features of DNA complexes with minor-groove binding drugs: (i) the drugs completely fill the minor groove and displace water in the AT-rich minor groove of DNA and (ii) the drugs protrude from the DNA and interact with neighbouring molecules. These findings demonstrate that further studies of oligonucleotides with different sequences are required in order to fully understand the structural features of the interaction of DNA with drugs. 

## Supplementary Material

PDB reference: d(AAAATTTT)_2_–CD27, 4ocd

Biological assays, DLS and supplementary figures.. DOI: 10.1107/S139900471400697X/dz5324sup1.pdf

## Figures and Tables

**Table 1:** Data-collection and refinement statistics Values in parentheses are for the highest resolution shell.

Data collection	
Beamline	BL13-XALOC, ALBA
Wavelength (Å)	0.97949
Resolution range (Å)	39–2.1 (2.21–2.10)
Space group	*P*6_1_22
Unit-cell parameters (Å, °)	*a* = *b* = 78.01, *c* = 91.55, α = β = 90, γ = 120
Total reflections	114934 (9823)
Unique reflections	10091 (1433)
Multiplicity	11.4 (6.9)
Completeness (%)	99.8 (99.8)
〈*I*/σ(*I*)〉	30.2 (3.0)
Wilson *B* factor (Å^2^)	59.64
*R*_merge_ †	0.036 (0.578)
Refinement	
*R*_work_ ‡/*R*_free_ §	0.2361/0.2510
No. of reflections	9432
No. of non-H atoms	622
DNA duplexes per asymmetric unit	1.5
Ligands	3 CD27
Waters	64
R.m.s.d., bond lengths (Å)	0.0238
R.m.s.d., angles (°)	1.6525
Average *B* factor (Å^2^)	60.32

† *R*_merge_ = 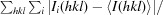 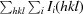.

‡ *R*_work_ and *R*_free_ were calculated as *R* = 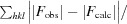 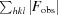.

§ *R*_free_ is the *R* factor evaluated for the reflections (5%) used for cross-validation during refinement.

**Table 2:** *In vitro* activity of CD27 and related analogues against *T. vaginalis*

		*T. vaginalis* †		*T. brucei rhodesiense* ‡	
		EC_50_ (µ*M*)		EC_50_ (µ*M*)	
Compound	Structure	Resorufin assay	Propidium iodide assay	Alamar Blue assay	*T*_m_ § (°C)
CD27	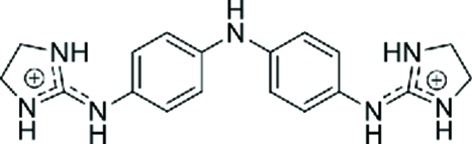	36.8 ± 6.9	25.1 ± 6.5	0.069	38.5
CD25	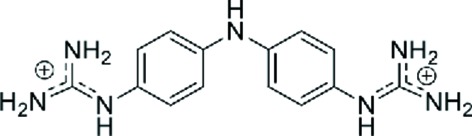	65.9 ± 9.7	48.7 ± 3.9	0.022	29.6
CD29	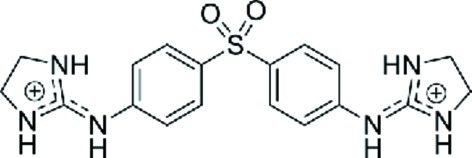	>100	71.7 ± 6.1	32.4	12.1
Reference drug	Metronidazole	0.66 ± 0.20	0.20 ± 0.03	—	

† *Trichomonas vaginalis* trophozoites of the metronidazole-susceptible strain G3.

‡ Bloodstream-form trypomastigotes of *Trypanosoma brucei rhodesiense* strain STIB900. These data have previously been reported (Dardonville & Brun, 2004 ▶; Rodríguez *et al.*, 2008 ▶) and are given here for comparative purposes.

§ Thermal melting for complexes with the poly(dAdT) oligonucleotide (Rodríguez *et al.*, 2008 ▶).

**Table 3:** Hydrogen bonds formed by CD27 in the minor groove of d(AAAATTTT)_2_ and external interactions with neighbouring phosphates All values are given in Å. A spatial representation of drugs *D* and *E* is shown in Figs. 2 ▶(*b*) and 3 ▶(*b*). The hydrogen bonds are ordered from the centre to the end of the duplex.

Atoms involved	Drug *D*	Drug *E*	Drug *F*
N3(A4)–N4(CD27)	3.06	2.88	2.77
O2(T6′)–N4(CD27)	3.08	3.24	3.26
N3(A2)–N3(CD27)	3.28	3.36	3.34
O2(T8′)–N3(CD27)	2.90	3.00	2.80
N1(CD27)–OP1(A2 oligo *B*′)	3.27	—	—
N7(CD27)–OP2(A3 oligo *B*′)	3.14	—	—
N7(CD27)–OP1(A3 oligo *B*′)	3.24	—	—
N2(CD27)–OP1(A3 oligo *B*′)	—	3.01	—

## Footnotes

1 Supporting information has been deposited in the IUCr electronic archive (Reference: DZ5324).
