# Supplementary material for: In and out of the minor groove: interaction of an AT-rich DNA with the drug CD27
Source: Acta Crystallogr D Biol Crystallogr. 2014 May 24;70(Pt 6):1614–21. doi: 10.1107/S139900471400697X (PMC4051503; doi:10.1107/S139900471400697X)
Supplement: Supplementary file 1 [file d-70-01614-sup1.pdf]

Supporting Information

In and Out the minor groove: Interaction of an AT-rich DNA with the CD27 drug

Francisco Acosta-Reyes, Christophe Dardonville, Harry P. de Koning, Manal Natto, Juan A. Subirana and J. Lourdes Campos

S1 Biological assays

Resorufin assay. *Trichomonas vaginalis* trophozoites of the metronidazole-susceptible strain G3 were cultured exactly as described previously in Diamond's medium with Hollander's modification (Hollander, 1976), supplemented with 10& heat-inactivated horse serum (Invitrogen). Cultures were grown anaerobically at 37 °C and passaged to fresh cultures every 24 h (max ~2×10^6 cells/ml). Test compound susceptibility was tested as described, using the fluorescent dye resorufin as a viability indicator (only live cells metabolize it to non-fluorescent dihydroresorufin), and metronidazole as a control drug. Briefly, test compounds were dissolved in DMSO at 20 mM and stored in aliquots; just before use 5 µl stock was diluted with 495 µl culture medium and 200 µl added to the first well of a 96-well plate. Of this 100 µl was taken and used for a serial doubling dilution over two rows of the plate, leaving the last well without drug as control. To each well, 100 µl cell suspension was added, to a final concentration of 5×10^4 trophozoites/well. Plates were tightly sealed with Nescofilm and incubated at 37 °C inside BD GasPak EZ pouches (BD Diagnostics, UK) to create anaerobic conditions. After 24 h, 20 µl of 125 µg/ml resazurin (Sigma) in sterile phosphate-buffered saline (PBS) was added to each well, followed by a further anaerobic incubation at 37 °C for 60 minutes, after which fluorescence was determined using a FLUOstar Optima (BMG Labtech GmbH, Offenburg, Germany) with filters at 544 nM (excitation) and 590 nm (emission). Half-maximum effect (EC_{50} values) was determined from non-linear regression, using the Prism 5.0 software package (GraphPad).

Propidium iodide assay. Propidium iodide (PI) assay for drug susceptibility was performed essentially as described above for the resorufin assay. Cells were grown

anaerobically at 37 °C on 96-well plates in the presence of doubling dilutions of test compound for 24 h, after which 30 µl of a solution containing 90 µM PI and 200 µM digitonin in PBS was added. After a further anaerobic incubation of 60 min at 37 °C fluorescence was read at 544 nm (excitation) and 620 (emission).

References

Hollander, D. H. (1976). *The Journal of parasitology* **62**, 826-828.

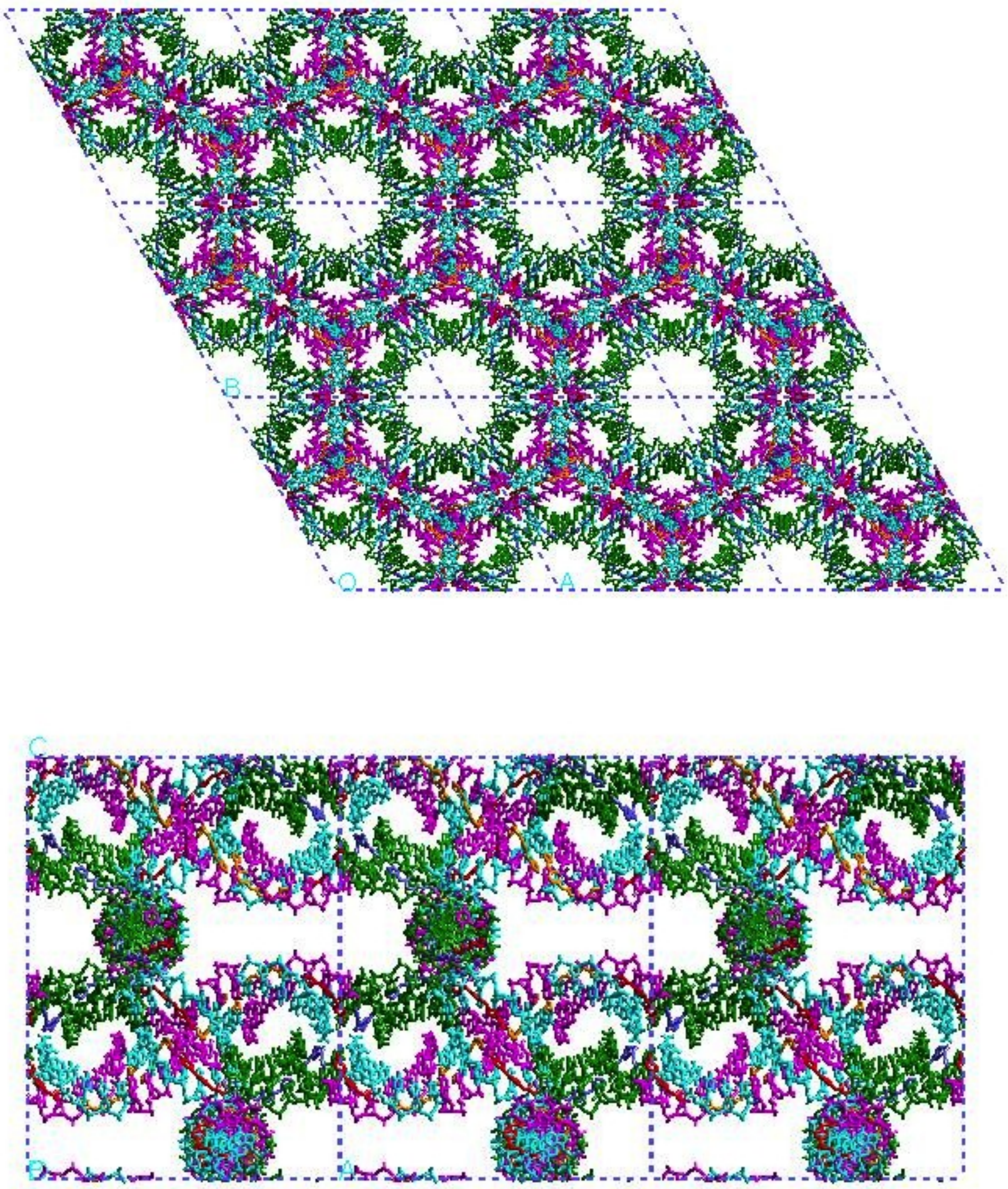

Fig. S1.1. Projection of nine unit cells along the z and y axis. DNA strands are colored as in Fig.2. The DNA duplex columns cross in space and leave large empty channels full of solvent. The crystal lattice is stabilized by DNA-drug-water contacts at the crossing points, as shown in detail in Fig. 3.

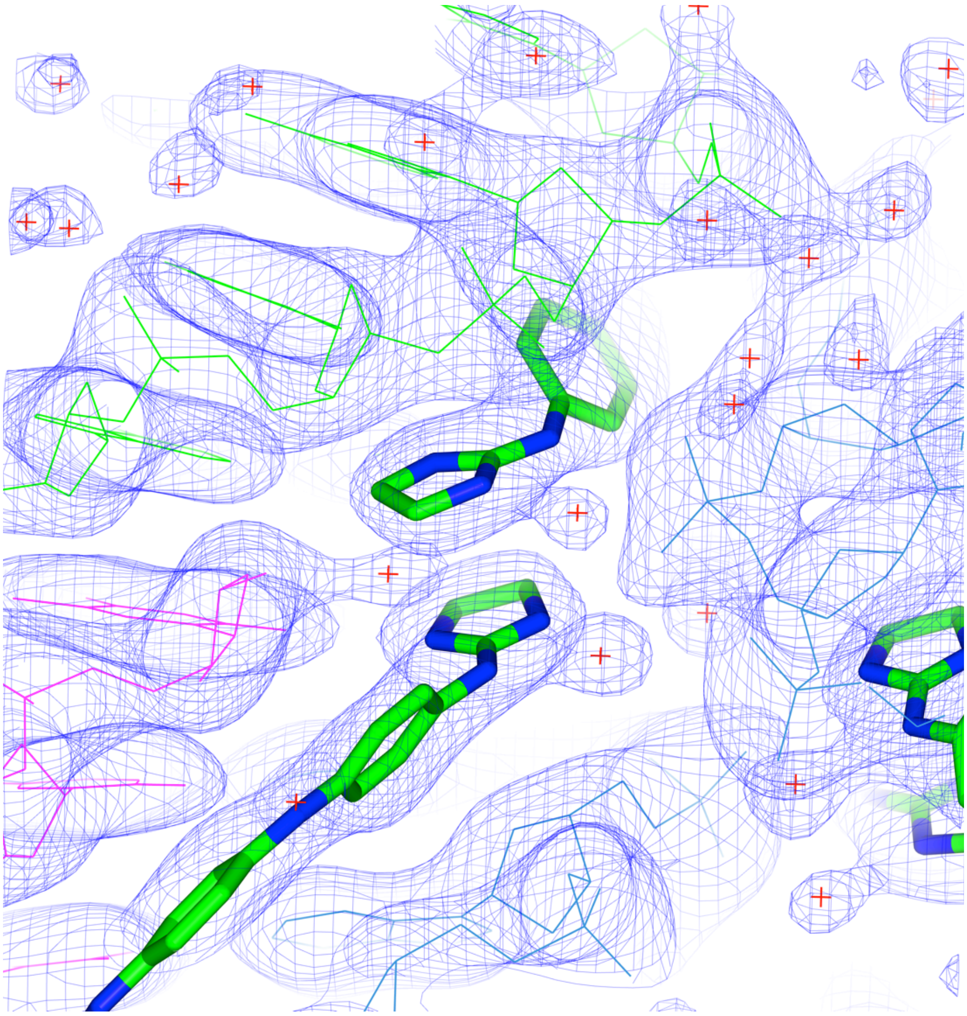

Fig. S1.2. Electron density map of the π-stacked ends of drugs D and E at 1σ level. Part of the surrounding DNA molecules is also shown. Nitrogen atoms of the drug are shown in dark blue. This figure corresponds to the region shown in Fig. 3b from a different point of view.

S2 Dynamic light scattering (DLS) studies on the influence of ions on the formation of drug-DNA crystals.

Dynamic light scattering is a powerful technique aimed to determine the dynamic aggregation of macromolecules, which are susceptible to crystallization. In our case we studied the size distribution and stability of oligonucleotides. Measurements were performed using a ZetaSizer Nano-S (Malvern Instruments Ltd.) red laser, 4mW, λ 633nm, with size detection between 0.3nm - 10μm. The backscattering angle used was 173º in all experiments, with a low volume quartz cell of 12-45 μL.

The set of measurements were done by monitoring size evolution at 13ºC when adding spermine, CD27, divalent ions and MPD to a DNA sample in sodium cacodylate buffer pH 6.5, in a total volume of 23 μL with the following final concentrations:

NaCac [mM]	DNA [mM]	Spm [mM]	CD27 [mM]	Ion [mM]	MPD [%]
32.5	0.086	0.035	0.22	3.12	8.7

*In the case of Zn the final concentration is 1.60 mM.

The drug-DNA ratio used is 2.5, which should allow an optimal interaction in the ratio 1:1 or 2:1 and the optimal interaction. Confirmed as the final drug ratio in the crystal structure is 2:1 (CD27:DNA).

The DNA sample in 40mM sodium cacodylate buffer showed a polydispersity index of about 0.6, which indicates the presence of species with large differences in size between them. This may be due to physical equilibrium, where a small amount of the sample is forming species with a larger size. The volume distribution shows a major species with hydrodynamic diameter of 2.25 nm (100%), which corresponds to single isolated oligonucleotide duplex molecules. The other non-representative specie is about 500 nm (< 0.001%). Upon addition of spermine the diameter increases to 2.60 nm, due to binding of the polycation.

Analysis of the influence of ions

Ions play an important role in charge neutralization of the negative charge of phosphate groups. We have monitored by DLS the size distribution of DNA upon addition of CD27 and divalent cations. We have thus determined the stabilization of the structure and its nucleation for the formation of multimeric species (nuclei), which are crucial in the crystallization step.

All measures were performed at the same concentrations as reported above, at 13°C using the oligonucleotide A_4T_4 , only changing the divalent ion. We used $Mn^{2+,}$ Mg^{2+},

Ca^{2+} and Zn^{2+}. Upon addition of ions the hydrodynamic diameter increased. Association complexes appeared upon addition of MPD, which acts as precipitant. Nucleation is associated with the formation of multimeric species, as shown in Table S2.1. The evolution of size distributions is shown below for two ions (Figures S2.1 and S2.2). Results for Ca^{2+} and Zn^{2+} are similar to those obtained with Mg^{2+} and are summarized in Figure S2.3 and Table S2.1. Manganese has the best proportion of peak size and percentage of multimeric species: it was used for our crystallization trials. The crystals obtained are shown in Figure S2.4.

A more detailed analysis of our DLS experiments and comparison with other oligonucleotides will be reported elsewhere.

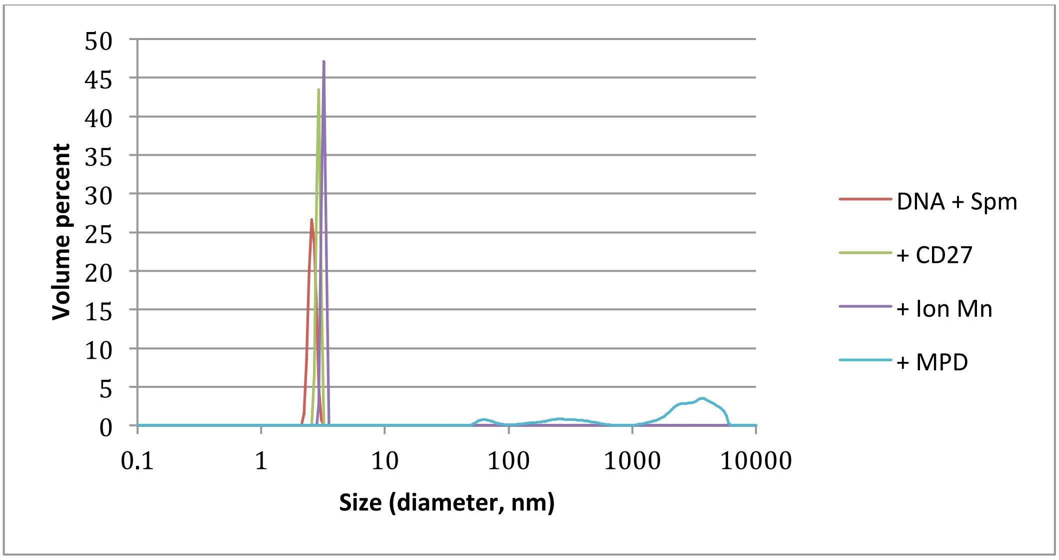

Figure S2.1. Evolution of size distribution for A_4T_4 + Spm + Cd27 + $MnCl_2$ + MPD

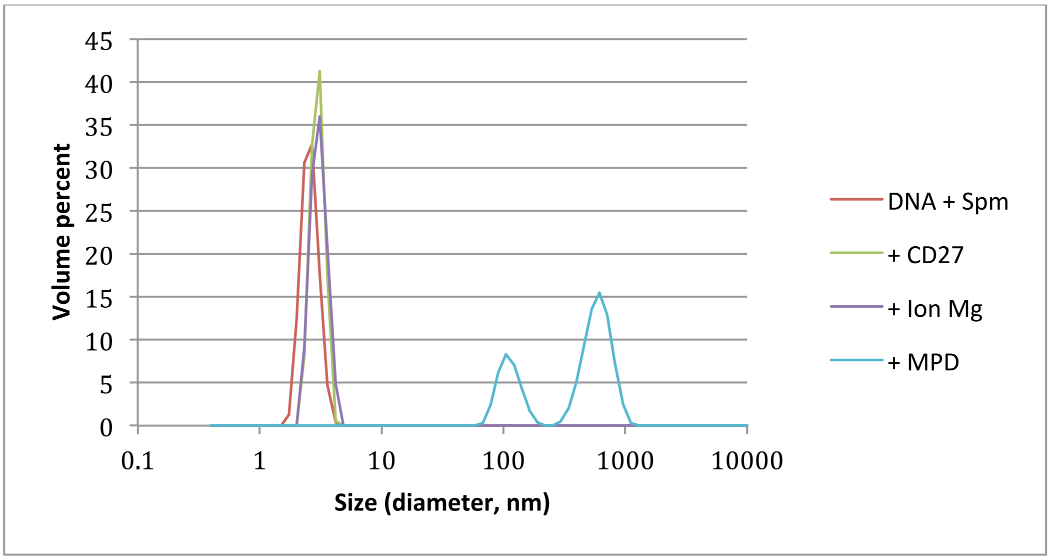

Figure S2.2. Evolution of size distribution for A_4T_4 + Spm + Cd27 + $MgCl_2$ + MPD

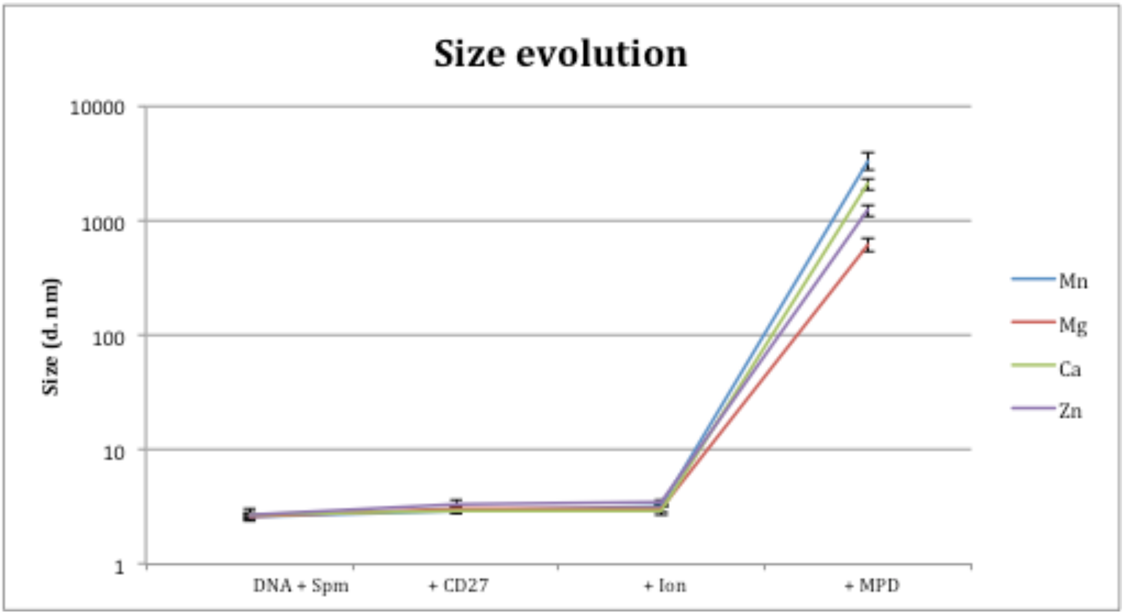

Figure S2.3. Size evolution of the major species for all ions.

Table S2.1. Final species distribution for all ions.

Ion	Peak 1	Peak 2	Peak 3	Peak 1	Peak 2	Peak 3
	Size [d.nm]	Size [d.nm]	Size [d.nm]	% volume	% volume	% volume
Mn^{2+}	3312	300.3	68.47	73.5	19.9	6.6
Mg^{2+}	606.1	113.4	0	69.2	30.8	0
Ca^{2+}	2074	245.9	79.8	88.1	7	4.9
Zn^{2+}	1221	350.4	111.6	70.8	16.1	13

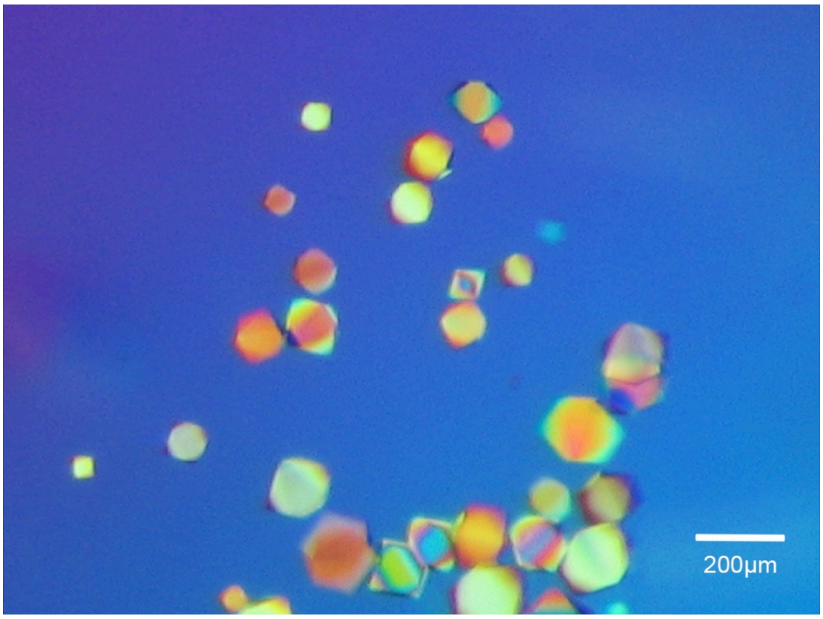

Fig. S2.4. Crystals used for X-ray crystallography, obtained in the presence of $MnCl_2$
